# Combined therapeutic option for NDM-producing *Serratia Marcescens* – an in vitro study from clinical samples

**DOI:** 10.1016/j.bjid.2024.104481

**Published:** 2024-11-26

**Authors:** Balbina Chilombo Albano, Leticia Ramos Dantas, Gabriel Burato Ortis, Paula Hansen Suss, Felipe Francisco Tuon

**Affiliations:** Pontifícia Universidade Católica do Paraná, Faculdade de Medicina, Laboratório de Doenças Infecciosas Emergentes, Curitiba, PR Brazil

**Keywords:** Serratia, Synergism, Carbapenemase, Aztreonam, Polymyxin

## Abstract

**Background:**

Treating NDM-producing bacteria poses a significant challenge, especially for those bacteria inherently resistant to polymyxin, such as *Serratia marcescens*, necessitating combined therapies.

**Objective:**

To assess in vitro the synergistic effect of different antimicrobial combinations against NDM-producing *S. marcescens*.

**Methods:**

Four clinical isolates were tested with various antibiotic combinations: polymyxin, amikacin, meropenem, and aztreonam. Concentrations used were those maximized by pharmacokinetic and pharmacodynamic assessments. Synergy evaluation involved a static macrodilution test followed by a time-kill curve assay.

**Results:**

All four isolates demonstrated resistance according to CLSI and EUCAST standards for the tested antibiotics (polymyxin, amikacin, meropenem, and aztreonam). In the macrodilution synergy test, the combination of aztreonam and amikacin was active in 2 out of 4 isolates within 24 h, and polymyxin with meropenem in only one isolate, despite of intrinsic resistance to polymyxin. However, time-kill curve analysis revealed no synergism or additive effect for combinations with the tested antimicrobials.

**Conclusion:**

Combinations of polymyxin, meropenem, aztreonam, and amikacin at doses optimized by pharmacokinetic/pharmacodynamic were insufficient to demonstrate any synergism in NDM-producing *S. marcescens* isolates in time-kill curves.

## Introduction

Among bacteria with high rates of Antimicrobial Resistance (AMR), enterobacteria are among the leading causes of infections in terms of case numbers, belonging to the ESKAPE group, responsible for the highest mortality rates from infections worldwide, designated as a “priority status” by the WHO.^1^ Enterobacterales are gram-negative microorganisms that ferment glucose, present in both natural environments and biological isolates, these microorganisms inhabit the gastrointestinal tract of humans, seamlessly integrating into the innate microbiota of these vital organs. Consequently, they serve as a conceivable reservoir for various pathogens.

In this group, *S. marcescens* has always warranted special attention as it is one of the causative agents of nosocomial infections that are difficult to treat. It exhibits intrinsic resistance to polymyxin B, ampicillin, and first and second-generation cephalosporins, necessitating the use of empirical antibiotic therapy to treat it. These bacteria have the ability to acquire resistance genes easily, with Metallo-β-Lactamases (MBL) being the most concerning.^2^ In Enterobacterales, the most important MBL is New Delhi Metallo-beta-lactamase (NDM), which incidence has increased in Brazil.^3^ Since the initial report, sporadic reports of NDM in other bacterial strains, including *S. marcescens*, have increased, rendering it Extensively Drug-Resistant (XDR).^4^

For infections caused by NDM-producing enterobacteria, polymyxin has been the main antibiotic in developing countries, as new carbapenemase inhibitors (relebactam, vaborbactam, avibactam) in combination with aztreonam are prohibitively expensive, making their use in public health unfeasible.^5^ The aim of this study was to assess in vitro the synergistic effect of different antimicrobial combinations against clinical strains of NDM-producing *S. marcescens* through static and dynamic analysis.

## Methods

### Setting

This study was approved by the local Ethical Committee (PUCPR, Curitiba, Brazil) with the number 74,239,517.3.2008.0020. The study was conducted at the Laboratory of Emerging Infectious Diseases of the Pontifícia Universidade Católica do Paraná using clinical isolates of NDM-producing *S. marcescens* identified between 2022 and 2023. A comprehensive assessment was conducted on a total of 10,684 carbapenem-resistant isolates belonging to *Enterobacterales, Pseudomonas* spp., and *Acinetobacter* spp. These isolates were sourced from various hospitals across eight cities in Southern Brazil. Upon reception, they were cultivated on McConkey agar, subjected to DNA extraction, and subsequently preserved at −80 °C in Brain Heart Infusion (BHI) broth supplemented with 10 % glycerol until further processing. Bacterial isolates were spotted on an inox slide with 1 uL of α-Cyano-4-hydroxycinnamic acid and identification was carried on using Matrix-Assisted Laser Desorption/Ionization-Time of Flight Mass Spectrometry (MALDI-TOF MS) system Vitek-MS (Biomérieux, Marcy L'Etoile, France). From this pool of microorganisms, all *S. marcescens* (four isolates) were selected after identification for further studies.

### DNA extraction and blaNDM identification

blaNDM gene extraction and amplification procedures involved heating microcentrifuge tubes containing a 0.5 McFarland bacterial inoculum in a dry thermoblock at 95 °C for 5 min. Following this, centrifugation was performed, and the resultant samples were stored in a freezer at −20 °C. Detection of blaNDM was accomplished utilizing the qPCR CDC protocol, as previously published.^4^ For the test assay, reaction mixtures of primers forward, reverse and probes (NDM-F_GACCGCCCAGATCCTCAA, NDM-R_CGCGACCGGCAGGTT and probe NDM_HEX-TGGATCAAGCAGGAGAT-BHQ1) plus MasterMix Taqman Fast (Applied Biosystems, Vilnus, Lithuania) and sterile reagent grade water (11 μL final volume) were mixed with 1 μL of crude lysate before analysis on 7500 Fast platform (Applied Biosystems, Carlsbad, CA).

### Synergism tests

Minimum Inhibitory Concentrations (MIC) were determined by broth microdilution according to CLSI standards (CLSI. Performance Standards for Antimicrobial Susceptibility Tests. 30th ed. Wayne, PA.: Clinical and Laboratory Standards Institute; 2020). A bacterial suspension with a standardized turbidity equivalent to 0.5 McFarland standard was used for tests, with 100 μL in each well. A series of two-fold dilutions of the antibiotics in Mueller-Hinton broth were add to each well. The ranging doses is described below. The microplates were incubated in appropriate temperature (37 °C) for 24 h. The MIC was defined by observing the lowest concentration of antibiotic that completely inhibits visible bacterial growth (absence of turbidity in the well. Polymyxin B sulfate (Haler chemical, Brazil) (from 0.0625 to 128 mg/L), amikacin sulfate (Teuto, Brazil) (from 1 to 128 mg/L), meropenem trihydrate (Eurofarma, Brazil) (from 0.25 to 128 mg/L), and aztreonam (Biochimico, Brazil) (from 1 to 128 mg/L) were utilized to determine the MIC. For the synergism test, the concentration of each antimicrobial was 2 mg/L of polymyxin, 8 mg/L of amikacin, 16 mg/L of meropenem, and 16 mg/L of aztreonam. All combinations of these antibiotics were tested.

For synergism test the bacterial inoculum was prepared to obtain a final concentration of ∼1 × 10^5^ cfu/mL using the 0.5 McFarland standard. The synergy test was conducted as previously described.^6^ Samples of clinical isolates were added to 6 tubes with different combinations of antibiotics and in 4 tubes with each individual antibiotic. A positive and negative control were included. The negative control was performed in a tube with Mueller Hinton broth only, and positive control include the isolated without antibiotic. Each tube presented 2 mL and were incubated at 37 °C for 24 h, and the result was assessed by visually analyzing turbidity. The presence of synergy is suggested when the tube exhibits no turbidity, resulting in a clear broth. Conversely, the absence of synergy is indicated by the presence of any turbidity observed in the tube.^6^

### Time-kill curve

In the in vitro time–kill assay, each isolate of S. marcescens was inoculated at a concentration of 5  ×  10^5^ cfu/mL into 10 mL of fresh CAMHB (Oxoid, Basingstoke, UK), followed by incubation at 35 °C with individual antibiotics and their combinations. Meropenem (16 mg/L), Amikacin (8 mg/L), Aztreonam (16 mg/L), and Polymyxin (2 mg/L) were tested both alone and in various combinations. At specified intervals (0, 1, 4, 12, and 24 h) post-inoculation, aliquots were extracted. These aliquots underwent serial dilution (10^–1^ to 10^–8^) and were plated in triplicate on TSA plates to facilitate colony counting. To ensure accuracy, potential antimicrobial carry-over was monitored by streaking transferred aliquots over agar plates, observing for any growth inhibition at the initial streak site. Time-kill curves were then constructed by correlating mean colony counts with time. Interpretation of results was conducted after 24 h of incubation, following established protocols.^7^

Data were descriptive, with colony count expressed as median and interquartile range. Logarithmic curves in the time. Time kill curve of each antibiotic and combination were compared with positive control and a reduction of 2 log were considered significant.

## Results

The four identified *S. marcescens* isolates were selected based on resistance to carbapenems, and a subsequent molecular test detected the blaNDM gene. The isolates exhibited resistance to all tested antimicrobials, including polymyxin, meropenem, aztreonam, and amikacin. The isolates were subjected to MIC testing using broth microdilution following CLSI guidelines (Table 1).

**Table 1:** Minimum inhibitory concentrations of amikacin, aztreonam, meropenem and polymyxin B alone against NDM-producing *S. marcescens* isolates and visual turbidity analysis from the synergism test in 24 and 48 h.

Antibiotic	Strain 7	Strain 24	Strain 25	Strain 32
	MIC			
Amikacin	128 mg/L	128 mg/L	128 mg/L	128 mg/L
Aztreonam	128 mg/L	32 mg/L	64 mg/L	32 mg/L
Meropenem	128 mg/L	128 mg/L	128 mg/L	128 mg/L
Polymyxin	128 mg/L	128 mg/L	128 mg/L	128 mg/L
	
**Combinations of antibiotics**	**24-hour readout**			
ZA	+	–	+	–
MA	–	–	–	–
MZ	–	–	–	–
PZ	–	–	–	–
PM	–	–	–	–
PA	+	–	–	–
	**48-hour readout**			
ZA	+	+	+	+
MA	+	+	+	–
MZ	+	–	+	–
PZ	+	–	+	–
PM	+	+	+	–
PA	+	+	+	+8h

The sign “+” indicates that turbidity was visible (possible absence of synergism), and “-” indicates that turbidity was not visible (possible synergism). ZA, Aztreonam + Amikacin; MA, Meropenem + Amikacin; MZ, Meropenem + Aztreonam; PZ, Polymyxin + Aztreonam; PM, Polymyxin + Meropenem; and PA, Polymyxin + Amikacin.

To assess synergy, two tests were conducted: first, a broth macrodilution test containing the individual antibiotics and their combinations. In the 24-hour macrodilution test, growth was observed in 2 out of 4 isolates with amikacin and aztreonam; meropenem and amikacin, meropenem and aztreonam, polymyxin and aztreonam, and polymyxin and meropenem showed no growth; polymyxin and amikacin showed growth in 1 out of 4 isolates. After a 48-hour reading, growth was observed in several combinations that were initially sensitive, as described in Table 1. This screening test was carried out with two reading times (24 h and 48 h), to understand the dynamics considering whether the effect in 24 h was bactericidal or bacteriostatic. However, the correct assessment of this interpretation requires the time-kill test, which was carried out sequentially.

**Fig. 1:**
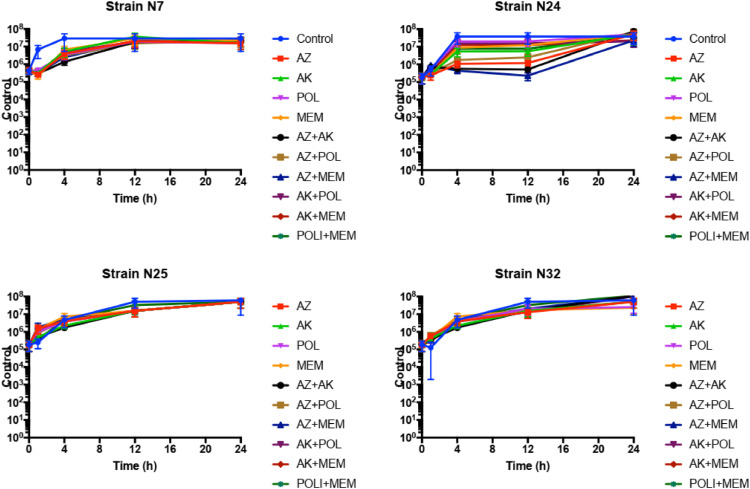
Time-kill curve of four isolates of *S. marcescens* exposed to different antibiotics and combinations. The blue line is the positive control. AZ, Aztreonam; AK, Amikacin; POL, Polymyxin; MEM, Meropenem.

In the evaluation of the time-kill curve for the four isolates, there was no significant reduction in growth, both with individual antimicrobials and their combinations (Fig. 1). In isolate N24, some combinations showed a 2-log drop compared to the control, but this drop was not considered bacteriostatic as it maintained growth from time zero, and in all combinations, there was regrowth within 24 h. This result may demonstrate that a synergism test with a 24 hour reading is not reliable, requiring more reliable tests. Furthermore, it brings into question other commercially available tests, knowing that we use a macrodilution test at adequate concentrations per PKPD.

## Discussion

In many countries around the world, particularly in developing nations, the new beta-lactamase inhibitors are not readily available, either due to cost-related issues or other constraints. This lack of availability renders it impossible to utilize them in combination with aztreonam, which has become the current treatment of choice of NDM-producing *S. marcescens*.^5^ This is the driving force behind our decision to assess the proposed antibiotics, aiming to evaluate synergism and provide a secondary treatment option. *Serratia marcescens* poses a formidable challenge in treatment when multidrug-resistant, as its intrinsic resistance to certain cephalosporins and polymyxins diminishes therapeutic options. Moreover, in cases where carbapenem resistance is present, therapeutic alternatives are further constrained to new beta-lactamase inhibitors (such as relebactam, avibactam, and vaborbactam).^6^ A previous study demonstrated that the in vitro combination of amikacin and meropenem exhibited a 68.4 % synergy in NDM-producing *Klebsiella pneumoniae* isolates.^8^ Similar results with E. coli has been observed.^9^ In our study, we observed a synergistic effect in *Serratia* spp.; however, after 48 h, there was a loss of antibiotic activity, revealing that the effect was bacteriostatic.

*Serratia marcescens* is intrinsically resistant to polymyxin due to electric charge of Lipopolysaccharides (LPS) of this microorganism. While meropenem may not directly affect the electrical charge of LPS, which constitute the outer layer of the cell membrane of *Serratia*, its antibacterial activity may indirectly impact membrane integrity and composition, including LPS expression or structure.^10^ In this aspect, a theorical synergism is possible. The combination of amikacin + meropenem, meropenem + colistin, and amikacin + colistin has already tested on 11 isolates of *K. pneumoniae* positive for the blaNDM gene.^11^ Of these combinations, the most effective was amikacin + meropenem, inhibiting 81.8 % of the tested strains. Meropenem + colistin was effective in 36.4 % of isolates, and amikacin + colistin was effective in 27.3 % of tested strains.^11^ Meropenem + colistin obtained excellent results in other studies, achieving a survival probability of approximately 80 % against an NDM-producing carbapenemase isolates.^11^ This combination has been shown to be effective in reducing MIC and consequently reducing medication side effects for the patient.^12^ The authors show that compared to other attempts at synergism, the test has excellent results, as all tested strains were resistant to at least one of these antibiotics, posing risks of resistance and high toxicity due to the high dosage.^11^ Several tests have determined that meropenem + colistin yield positive results against NDM strains.^13^ In our study, we have also observed synergism, however, in the TKC, this find was not sustainable.

The combination of amikacin and colistin has been tested in vitro against several NDM-producing Enterobacteriaceae strains, demonstrating a synergistic effect in 27.3 % of cases. In these studies, the authors randomly selected one NDM strain and conducted a time-kill assay with this combination, revealing a bacteriostatic effect.^11^ This result was not obtained in our study, suggesting that these combinations cannot be extended to *S. marcescens*. In the literature, aztreonam-colistin has not shown efficacy in NDM. Comparing the results with our study, there was a bacteriostatic action, inhibiting consistent growth within 24 h, but there was growth in 2 strains at 48 h. The time-kill curve demonstrated that the combination was ineffective in vitro.^8^

MBLs are enzymes that can be inhibited by aztreonam, and the rationale for combinations with aztreonam, even with high MIC, is that the concentration of Mg and Zn in tissues is lower than that used in vitro tests, suggesting the maintenance of activity and potential clinical use.^14^ These facts are important before extrapolate in vitro studies to in vivo. However, if an in vitro study demonstrate synergism, the potential clinical use overhang.

This study is limited by the number of isolates tested, the in vitro evaluation which cannot be extrapolated to real life, and potential clonality. We did not evaluate clonality because the isolates originated from different cities located >300 kms apart. Although distance is not necessarily indicative of clonality, we believe it is unlikely to be a factor.

In conclusion, our study demonstrated that static synergy testing (in tubes), despite showing synergism, did not confirm this in dynamic testing, indicating that in these isolates, the test not only lacked validity but also showed no effective combination. In summary, static in vitro tests did not correlate with dynamic tests, and it is not possible to confirm that combinations of polymyxin, meropenem, amikacin, and aztreonam are effective against NDM-producing *S. marcescens*. Given the extensive antimicrobial resistance exhibited by NDM-producing bacteria, there are growing challenges in treatment, especially in the absence of new drugs with robust activity. In clinical practice within our country, combination therapy remains the most frequently employed strategy by physicians to combat these resistant microorganisms.

## Funding

This research received no external funding.

## Authors’ contributions

Conceptualization, F.T.; methodology, L.D and GO and PS.; formal analysis, F.T.; investigation, B.A.; resources, F.T.; writing-original draft preparation, G.O. and F.T.; writing-review and editing, P.S.; visualization, F.T. All authors have read and agreed to the published version of the manuscript.

## Informed consent statement

Not applicable.

### Data availability statement

Data are available under request.

## Conflicts of interest

The authors declare no conflicts of interest.
